# Physical Activity Level and Risk of Death: The Severance Cohort Study

**DOI:** 10.2188/jea.JE20110110

**Published:** 2012-11-05

**Authors:** Yejin Mok, Soyoung Won, Heejin Kimm, Chungmo Nam, Heechoul Ohrr, Sun Ha Jee

**Affiliations:** 1 Institute for Health Promotion, Department of Epidemiology and Health Promotion, Graduate School of Public Health, Yonsei University, Seoul, Korea; 2 Department of Preventive Medicine and Public Health, College of Medicine, Yonsei University, Seoul, Korea

**Keywords:** physical activity, cancer, death, metabolic equivalent of task

## Abstract

**Background:**

Physical activity decreases deaths from cardiovascular disease and other causes; however, it is unclear whether physical activity is associated with cancer incidence and death in Asian populations.

**Methods:**

Data from 59 636 Koreans aged 30 to 93 years were collected using a questionnaire and medical examination at the Severance Hospital Health Promotion Center between 1994 and 2004. Study participants were followed for a mean duration of 10.3 years.

**Results:**

In the exercising group, the multivariate Cox proportional hazards model showed a lower risk of cancer death (hazard ratio [HR] = 0.72, 95% CI = 0.62–0.85) in men but not in women. Those who exercised, as compared with those who did not, had lower risks of all-cause death (men: HR = 0.68, 95% CI = 0.60–0.76; women: HR = 0.65, 95% CI = 0.53–0.79) and noncancer death (men: 0.63, 0.53–0.75; women: 0.52, 0.39–0.69). Physical activity was inversely associated with risk of noncancer death among men and women.

**Conclusions:**

Physical activity was associated with lower risks of cancer death and noncancer death.

## INTRODUCTION

Regular physical activity prevents obesity,^1^ improves cardiovascular risk factors,^2^^,^^3^ and reduces cardiovascular disease (CVD) incidence and mortality.^4^ Physical activity is also associated with a reduction in all-cause deaths.^5^^,^^6^ Although some research indicates that only vigorous activity reduces the risk of chronic health conditions,^7^ this is controversial. Studies have found a dose–response relationship,^8^ as well as reverse J-shape,^9^ L-shape,^10^ J-shape, and U-shape^11^ associations, between physical activity and all-cause death. Cancer is now the leading cause of death in South Korea. Between 1999 and 2009, cancer deaths increased from 114.2 to 140.5 deaths per 100 000 person-years in Korean adults.^12^ According to recent reports, physical activity has beneficial effects on cancer risk.^13^ However, most studies of the benefits of exercise have focused on death from CVD^5^^,^^6^^,^^10^^,^^14^^–^^17^ rather than cancer risk^13^^,^^15^^–^^17^ or cancer mortality,^5^^,^^6^^,^^10^^,^^14^^–^^16^ and most studies have investigated Western populations. Large-scale follow-up studies have been conducted in only 2 Asian countries.^13^^,^^18^

Metabolic equivalent of task (MET)-min/week calculated from walking, moderate physical activity, and vigorous physical activity^19^ is a relatively accurate measure of physical activity.^20^ However, it would be more useful for predicting individual health status if simple MET-min/week values were calculated based on a few types of common physical activities. Therefore, the purpose of this study was to use simple MET-min/week calculations from 9 common physical activities to determine the association of physical activity with cancer incidence and mortality, using data collected from participants enrolled from 1994 through 2004 at the Severance Hospital Health Promotion Center.

## METHODS

### Study participants

The cohort consisted of 70 862 Korean men and women who ranged in age from 20 to 93 years and participated in at least 1 medical evaluation at the Severance Hospital Health Promotion Center between 1994 and 2004. To avoid the confounding effect of a pre-existing condition on the association of physical activity with death, we excluded 4323 participants younger than 30 years, 119 participants who reported having any cancer, 687 participants who reported having cardiovascular disease, and 327 participants who reported having lung disease or asthma. In addition, we excluded 4695 participants with missing information on smoking status or alcohol intake and 95 participants with missing exercise information. The final study sample included 59 636 participants. Written consent was not obtained from participants before 2005. However, after 2004 such consent was obtained from all participants after nurses explained the purpose of the study. The Yonsei University Institutional Review Board on Human Research approved this study.

### Data collection

Participants were asked to describe their smoking habit (never smoker, ex-smoker, or current smoker), alcohol consumption (nondrinker or drinker of any amount of alcohol), and demographic characteristics such as age, sex, and past history of diabetes or hypertension. Body weight and height were measured while participants were wearing light clothing. A registered nurse or technician measured blood pressure (BP) using a standard mercury sphygmomanometer while participants were in a seated position. Systolic and diastolic BP was measured after a minimum 5-minute rest period.

### Measurement of biomarkers

For the clinical chemistry assay, serum was separated from peripheral venous blood samples that were obtained from each participant after a minimum of 12 hours of fasting and stored at −70°C. Biomarkers of metabolic syndrome such as fasting blood glucose, total cholesterol, high-density lipoprotein (HDL) cholesterol, and triglyceride levels were measured using a Hitachi 7600 analyzer (Hitachi Ltd., Tokyo, Japan). All measurements were performed by the central laboratory at Severance Hospital, Yonsei University Health System, Seoul, Korea. Data quality was maintained in accordance with the procedures of the Korean Association of Laboratory Quality Control.

### Assessment of physical activity level

The main parameter in the present study was physical activity level. In our survey, 4 questions on physical activity were asked during the baseline evaluation. The first question was “Do you exercise regularly?”. The possible responses were “yes” and “no”. If participants answered “yes”, they went on to the next question, which was “If you exercise, which of the following 9 types of physical activity do you do: jogging, rope jumping, walking, mountain climbing, calisthenics, swimming, yoga, aerobics, or golf?”. The next question asked the average amount of time spent (minutes per exercise) exercising and what physical activity (of 9 options) was frequently done during the week. Each activity on the questionnaire was assigned a MET value,^21^ which was calculated based on the energy expended by sitting quietly. Next, we multiplied the MET value of each activity by the average amount of time spent exercising (minutes per exercise) and by frequency of exercise per week. Finally, we calculated MET-min/week to estimate physical activity levels.

### Follow-up and outcomes of interest: cancer incidence, all-cause death, cancer death, and noncancer death

Participants were followed from their starting point until 31 December 2010. Cancer incidence was identified via notification from the National Cancer Center until 31 December 2008. We calculated total cancer incidence after identifying participants who received a diagnosis of any cancer according to data from the Korean National Cancer Center. Computerized searches for death certificates were performed until 31 December 2009, using the identification number assigned at birth by the National Statistical Office. Because cause of death was not included in our data, we used the category noncancer death, which was defined as the difference between all-cause deaths and cancer deaths.

### Statistical analysis

We divided our study samples into 3 groups based on physical activity level (0, 3.5–1000, or >1000 MET-min/week). Additionally, we created a category for diabetes by combining participants with self-reported antidiabetic treatment or a fasting serum glucose level of 126 mg/dL or higher. Hypertension was defined as a systolic BP of at least 140 mm Hg, a diastolic BP of at least 90 mm Hg, or self-reported antihypertensive treatment.

Multiple regression analyses were used to examine the association between physical activity level and cardiovascular risk factors at baseline. Also, we examined the association of physical activity level with cancer incidence, all-cause death, cancer death, and noncancer death. Cox proportional hazards models were examined after adjusting for age and other potential confounding factors, including smoking status, alcohol consumption, body mass index, hypertension, diabetes, and dyslipidemia. To avoid the possible confounding effects of health status on physical activity, we excluded participants who received a cancer diagnosis or died within 3 years of the starting point. Cox proportional hazards models were used to estimate the risk of cancer and death from all causes, cancer, and noncancer, after these exclusions. All analyses were performed separately for men and women, using SAS statistical software version 9.1 (SAS Institute Inc., Cary, NC, USA). All statistical tests were 2-sided, and statistical significance was defined as a *P* value less than 0.05.

## RESULTS

### Cohort characteristics

The characteristics of the participants by MET-min/week are shown in Table 1. The group with no physical activity accounted for 69.6% of the 59 636 (men: 50.7%, women: 49.3%) participants. Mean age and activity level were higher among physically active participants than among those who reported no physical activity. Those who were more physically active were more likely to have higher mean levels of total cholesterol and HDL cholesterol and less likely to have abnormal diastolic BP, γ-glutamyl transferase (GGT), aspartate aminotransferase (AST), and alanine aminotransferase (ALT) values. Also, physically active participants were less likely to be smokers. Men who were more physically active were more likely to have hypertension and type 2 diabetes. Women who were more physically active were less likely to have hypertension, but there was no significant difference in the prevalence of type 2 diabetes with respect to activity level.

**Table 1.** Baseline characteristics of study subjects according to MET-min/week (*n* = 59 636): The Severance Cohort Study, 1994–2004

	MET-min/week			*P* for trend
	
	0	3.5–1000	>1000	
**Men**	*n* = 21 051	*n* = 5808	*n* = 4805	

	mean ± SD	mean ± SD	mean ± SD	
	
Age (years)	45.6 ± 10.6	47.2 ± 10.0	49.4 ± 10.0	<0.0001
Body mass index (kg/m^2^)	24.1 ± 3.0	24.3 ± 2.7	24.5 ± 2.6	<0.0001
Systolic blood pressure (mm Hg)	124.0 ± 18.2	124.3 ± 18.1	125.9 ± 18.9	<0.0001
Diastolic blood pressure (mm Hg)	73.3 ± 12.0	73.0 ± 12.2	72.5 ± 12.1	<0.0001
Total cholesterol (mg/dL)	196.4 ± 35.5	197.7 ± 34.2	198.6 ± 34.0	<0.0001
HDL cholesterol (mg/dL)	46.2 ± 11.6	46.7 ± 11.5	47.7 ± 11.6	<0.0001
Triglyceride (mg/dL)	170.1 ± 118.4	164.4 ± 109.0	160.6 ± 114.1	<0.0001
Fasting glucose (mg/dL)	98.2 ± 26.5	98.5 ± 24.0	99.6 ± 25.1	0.0017
GGT (IU/L)	47.8 ± 68.3	40.1 ± 43.0	40.4 ± 42.3	<0.0001
AST (IU/L)	23.9 ± 23.7	22.1 ± 11.6	22.1 ± 10.8	<0.0001
ALT (IU/L)	30.0 ± 31.3	27.0 ± 21.6	25.5 ± 17.8	<0.0001

	%	%	%	
	
Smoking status				
Ex-smoker	24.3	34.0	40.3	
Current smoker	56.4	42.5	39.5	<0.0001
Alcohol intake (yes)	81.3	86.4	85.7	<0.0001
Hypertension (yes)	24.5	26.0	29.0	<0.0001
Diabetes (yes)	6.8	8.0	9.4	<0.0001

**Women**	*n* = 20 481	*n* = 3889	*n* = 3602	

	mean ± SD	mean ± SD	mean ± SD	
	
Age (years)	47.4 ± 10.8	48.3 ± 10.2	47.5 ± 9.2	0.0398
Body mass index (kg/m^2^)	23.5 ± 3.3	23.4 ± 3.0	23.6 ± 2.8	0.7922
Systolic blood pressure (mm Hg)	121.8 ± 21.0	121.1 ± 20.4	120.9 ± 19.5	0.0042
Diastolic blood pressure (mm Hg)	72.8 ± 12.0	71.5 ± 11.7	70.7 ± 11.7	<0.0001
Total cholesterol (mg/dL)	195.5 ± 37.9	197.6 ± 36.8	195.9 ± 36.0	0.0931
HDL cholesterol (mg/dL)	52.9 ± 12.8	54.8 ± 13.2	56.3 ± 13.6	<0.0001
Triglyceride (mg/dL)	125.8 ± 83.0	120.8 ± 81.0	113.8 ± 74.1	<0.0001
Fasting glucose (mg/dL)	94.4 ± 22.9	93.0 ± 18.6	92.8 ± 19.7	<0.0001
GGT (IU/L)	19.5 ± 27.1	18.2 ± 18.5	17.8 ± 16.4	<0.0001
AST (IU/L)	19.7 ± 17.0	19.1 ± 10.5	19.1 ± 8.9	0.0086
ALT (IU/L)	18.5 ± 22.0	17.8 ± 18.9	17.3 ± 12.7	0.0002

	%	%	%	
	
Smoking status				
Ex-smoker	2.3	2.6	2.4	
Current smoker	6.0	4.1	4.5	<0.0001
Alcohol intake (yes)	27.4	27.7	30.8	0.0001
Hypertension (yes)	25.5	24.5	22.7	0.0002
Diabetes (yes)	4.9	5.3	4.6	0.6578

MET: metabolic equivalent of task; SD: standard deviation; HDL: high-density lipoprotein; GGT: γ-glutamyl transferase; AST: aspartate aminotransferase; ALT: alanine aminotransferase.

As physical activity increased, the β-coefficient for diastolic BP and ALT significantly decreased, and that for HDL cholesterol significantly increased, in men and women (Figure).

**Figure.**
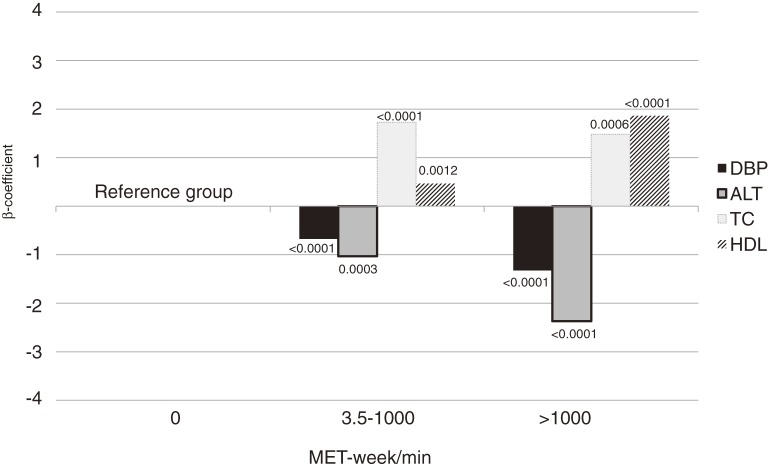
Regression β-coefficients of DBP, TC, HDL, and ALT according to MET-week/min. DBP: diastolic blood pressure; TC: total cholesterol; HDL: high-density lipoprotein cholesterol; ALT: alanine aminotransferase; MET: metabolic equivalent of task.

### Physical activity and death

Table 2 shows the hazard ratios (HRs) of cancer incidence and death according to MET-min/week in men. As MET-min/week increased, all-cause deaths and noncancer deaths gradually decreased as compared with no physical activity. This linear decrease in deaths according to MET-min/week was unchanged in additional analysis that excluded deaths occurring within 3 years of the starting point.

**Table 2.** Hazard ratios (95% CI) of cancer incidence, all-cause death, cancer death, and noncancer death by MET-min/week in men^a^

	MET-min/week				≥3.5 vs 0 MET-min/week
	
	0	3.5–1000	>1000	*P* for trend	
	(*n* = 21 051)	(*n* = 5808)	(*n* = 4805)		
**Cancer incidence**					
Cases	1308	364	340		
Rate^b^	9.3	8.9	8.9		
Model 1	1.0	0.92 (0.81–1.04)	0.89 (0.79–1.01)	0.0465	0.90 (0.82–1.00)
Model 2	1.0	1.00 (0.84–1.13)	0.98 (0.84–1.13)	0.0030	0.99 (0.88–1.11)

**All-cause death**					
Cases	1083	219	214		
Rate^b^	8.2	5.6	6.8		
Model 1	1.0	0.70 (0.60–0.81)	0.66 (0.56–0.76)	0.0013	0.68 (0.60–0.76)
Model 2	1.0	0.73 (0.62–0.87)	0.66 (0.55–0.79)	0.0081	0.70 (0.61–0.79)

**Cancer death**					
Cases	539	99	122		
Rate^b^	3.9	2.7	3.3		
Model 1	1.0	0.66 (0.53–0.81)	0.79 (0.64–0.96)	0.1818	0.72 (0.62–0.85)
Model 2	1.0	0.72 (0.57–0.92)	0.78 (0.62–0.98)	0.4211	0.75 (0.63–0.90)

**Noncancer death**					
Cases	544	120	92		
Rate^b^	4.3	2.9	3.5		
Model 1	1.0	0.74 (0.60–0.91)	0.52 (0.41–0.66)	0.0016	0.63 (0.53–0.75)
Model 2	1.0	0.75 (0.59–0.95)	0.54 (0.41–0.70)	0.0036	0.64 (0.53–0.78)

^a^Adjusted for age, smoking status, alcohol intake, body mass index, hypertension, total cholesterol, and diabetes.

^b^Age-adjusted rate (per 100 000 person years).

Model 1: Without exclusion of early cases (those occurring within 3 years of the starting point).

Model 2: After exclusion of early cases (those occurring within 3 years of the starting point).

Table 3 shows the HRs for cancer incidence and death according to MET-min/week in women. The results for women were similar to those for men, but physical activity appeared to have a greater effect on noncancer death among women. The HR for all-cause death among moderately active women (3.5–1000 MET-min/week) as compared with physically inactive participants was 0.59 (95% CI, 0.44–0.79). The HR for noncancer death was 0.56 (0.38–0.84) among women who were moderately physically active.

**Table 3.** Hazard ratios (95% CI) of cancer incidence, all-cause death, cancer death, and noncancer death by MET-min/week in women^a^

	MET-min/week				≥3.5 vs 0 MET-min/week
	
	0	3.5–1000	>1000	*P* for trend	
	(*n* = 20 481)	(*n* = 3889)	(*n* = 3602)		
**Cancer incidence**					
Cases	1139	211	193		
Rate^b^	6.7	6.7	6.7		
Model 1	1.0	0.93 (0.79–1.10)	1.02 (0.86–1.20)	0.5831	0.97 (0.86–1.10)
Model 2	1.0	0.94 (0.77–1.13)	0.99 (0.81–1.19)	0.3085	0.96 (0.83–1.11)

**All-cause death**					
Cases	630	81	66		
Rate^b^	5.0	3.2	4.4		
Model 1	1.0	0.60 (0.46–0.77)	0.71 (0.54–0.93)	<0.0001	0.65 (0.53–0.79)
Model 2	1.0	0.59 (0.44–0.79)	0.78 (0.58–1.04)	<0.0001	0.67 (0.54–0.83)

**Cancer death**					
Cases	235	27	38		
Rate^b^	1.6	1.1	1.5		
Model 1	1.0	0.62 (0.41–0.92)	1.07 (0.76–1.52)	0.1892	0.82 (0.62–1.08)
Model 2	1.0	0.62 (0.40–0.97)	1.17 (0.80–1.69)	0.3829	0.86 (0.64–1.17)

**Noncancer death**					
Cases	395	54	28		
Rate^b^	3.4	2.1	2.9		
Model 1	1.0	0.58 (0.41–0.82)	0.44 (0.28–0.69)	<0.0001	0.52 (0.39–0.69)
Model 2	1.0	0.56 (0.38–0.84)	0.47 (0.29–0.78)	<0.0001	0.52 (0.38–0.72)

^a^Adjusted for age, smoking status, alcohol intake, body mass index, hypertension, total cholesterol, and diabetes.

^b^Age-adjusted rate (per 100 000 person years).

Model 1: Without exclusion of early cases (those occurring within 3 years of the starting point).

Model 2: After exclusion of early cases (those occurring within 3 years of the starting point).

## DISCUSSION

In this cohort study, physical activity was associated with cancer incidence, all-cause death, cancer death, and noncancer death. Greater physical activity had a positive effect on noncancer deaths and all-cause deaths among both men and women.

In this study, men who were more physically active were more likely to have hypertension and type 2 diabetes, which suggests that highly physically active men have a greater chance of having a diagnosis of hypertension and type 2 diabetes. This could be the result of reverse causality in this cross-sectional study, ie, men newly diagnosed as having hypertension or type 2 diabetes might increase their physical activity, while women already exercise and have a low chance of being diagnosed as having hypertension or type 2 diabetes before menopause.

We found that physical activity was associated with diastolic BP, HDL, and ALT (Figure). Regular physical activity is known to independently increase HDL cholesterol^22^ and decrease BP.^23^ Also, physical activity is inversely associated with ALT level.^24^ Elevated ALT is associated with metabolic syndrome, independent of insulin resistance,^25^ and predicts CVD.^26^

We found that physical activity was inversely associated with all-cause death. Many studies have examined the association between physical activity and all-cause death^8^^–^^11^ and reported an inverse dose–response relationship between them^8^; however, some studies have shown a reverse J-shaped,^9^ L-shaped,^10^ J-shaped, or U-shaped^11^ curve. We found that physical activity was associated with a slight inverse dose–response relationship. The findings were similar after excluding deaths and cases of new cancer within 3 years of the starting point.

Physical activity was associated with cancer death only among men. However, the relationship between physical activity and cancer incidence was not significant. The effect of physical activity on cancer differs by cancer site.^27^ Therefore, our results may not be related to total cancer incidence. Those who regularly exercised had a lower risk of cancer death as compared with those who did not exercise. Only a few studies have explored the association between cancer risk and physical activity.^13^^,^^28^^,^^29^

The mechanisms by which physical activity affects cancer development at different sites are similar.^13^ The insulin/insulin-like growth factor (IGF) axis influences cell proliferation, differentiation, and apoptosis,^30^ and IGF-1 has an important role in carcinogenesis.^31^ Physical activity increases insulin sensitivity and decreases C-peptide levels, which improves insulin resistance.^32^ Thus, regular physical activity may influence cancer incidence and death via the IGF axis.

We found a significant inverse association between physical activity and risk of noncancer death. In Korea, cancer is the leading cause of death, followed by CVD.^12^ Therefore, our results may be related to the number of CVD deaths and can be explained by the fact that physical activity was positively correlated with HDL and inversely correlated with DBP and ALT in this study. The association between physical activity and CVD is well-known. However, the intensity of physical activity that is needed to prevent CVD is unclear. In some studies, only vigorous physical activity reduced CVD risk,^33^^,^^34^ while other studies suggested that non-vigorous physical activity was sufficient to reduce CVD risk.^35^^,^^36^ The present study found that physical activity was inversely associated with noncancer death. After exclusion of early deaths (those within 3 years of the study start), the results were unchanged. Previous studies have suggested that physical activity had an effect on CVD risk factors such as total, HDL, and LDL cholesterol and triglyceride levels.^37^^,^^38^

There have been few follow-up studies of large Asian populations. One such study found an association between cancer incidence and physical activity in Japan.^13^ That study assessed various activity categories, such as leisure-time and non-leisure time activity (occupational activity and housework). In the present study, we assessed physical activity by asking participants to choose only among leisure activities. Although the methods of assessment differed between the 2 studies, both found that physical activity was inversely associated with cancer risk.

The present study design was prospective, and the large study population was selected from the general population of Korea. An important limitation of the present study was that assessment of physical activity was based on self-reports; therefore, some misclassification was unavoidable. Also, on our questionnaire, only 1 response was checked for type of physical activity, which may have led to an underestimation of the associations. We do not have validation data for our calculation of MET-min/week. This is relevant because participants tend to over-report their physical activity, including duration and frequency of physical activity performed on a given day, which might have resulted in overestimation of the true relationship.^39^

In conclusion, physical activity affects cancer incidence, all-cause death, cancer death, and noncancer death. Physical activity was inversely associated with noncancer death and all-cause death in both men and women. There is a need for further research on the association of physical activity with the risks of specific cancers and death in the general population. In addition, optimal activity levels should be determined in mechanistic studies of physical activity intensity.
